# A role for ABCB1 in prognosis, invasion and drug resistance in ependymoma

**DOI:** 10.1038/s41598-019-46700-z

**Published:** 2019-07-16

**Authors:** Durgagauri H. Sabnis, Lisa C. D. Storer, Jo-Fen Liu, Hannah K. Jackson, J. P. Kilday, Richard G. Grundy, Ian D. Kerr, Beth Coyle

**Affiliations:** 1 Children’s Brain Tumour Research Centre, School of Medicine, University of Nottingham, Nottingham, UK; 2 Royal Manchester Children’s Hospital, Children’s Brain Tumour Research Network & Institute of Cancer Sciences, The University of Manchester, Manchester, UK; 3 School of Life Sciences, University of Nottingham, Nottingham, UK

**Keywords:** Cancer therapeutic resistance, Paediatric cancer, Tumour biomarkers

## Abstract

Three of the hallmarks of poor prognosis in paediatric ependymoma are drug resistance, local invasion and recurrence. We hypothesised that these hallmarks were due to the presence of a sub-population of cancer stem cells expressing the multi-drug efflux transporter ABCB1. *ABCB1* gene expression was observed in 4 out of 5 paediatric ependymoma cell lines and increased in stem cell enriched neurospheres. Functional inhibition of ABCB1 using vardenafil or verapamil significantly (p ≤ 0.05–0.001) potentiated the response to three chemotherapeutic drugs (vincristine, etoposide and methotrexate). Both inhibitors were also able to significantly reduce migration (p ≤ 0.001) and invasion (p ≤ 0.001). We demonstrate that ABCB1 positive patients from an infant chemotherapy-led trial (CNS9204) had a shorter mean event free survival (EFS) (2.7 versus 8.6 years; p = 0.007 log-rank analysis) and overall survival (OS) (5.4 versus 12 years; p = 0.009 log-rank analysis). ABCB1 positivity also correlated with reduced event free survival in patients with incompletely resected tumours who received chemotherapy across CNS9204 and CNS9904 (a radiotherapy-led SIOP 1999-04 trial cohort; p = 0.03). ABCB1 is a predictive marker of chemotherapy response in ependymoma patients and vardenafil, currently used to treat paediatric pulmonary hypertension in children, could be repurposed to reduce chemoresistance, migration and invasion in paediatric ependymoma patients at non-toxic concentrations.

## Introduction

Ependymomas are the second most common malignant brain tumour in children. Ependymal tumours occur across all age groups, but the outcome for children (67% 10 year overall survival (OS) age 0–19) is lower than in their adult counterparts (85–89% 10 year OS age 20–64)^1^. The poorest survival is seen in infants, with the 5 year survival standing at a bleak 42–55%^2^. The only clinical factor consistently associated with survival is the extent of surgical resection^3^, with even histopathological grading unable to reliably predict outcome, in part due to tumour heterogeneity^4^. These tumours occur throughout the central nervous system (CNS), but the majority of paediatric ependymomas are intracranial with over two-thirds arising in the posterior fossa (PF) whereas one-third occur supratentorially (ST)^5^. Despite recent molecular classification of ependymomas there remain only 2 documented biomarkers correlated with poor outcome, namely gain of the long arm of chromosome 1^6,7^, and the presence of RELA driver gene fusions in a subgroup of ST ependymomas^8,9^. To date there are no biological marker dependent treatments for ependymoma.

Current treatment protocols for paediatric ependymoma combine surgical resection with a combination of radiotherapy and chemotherapy. Radiotherapy treatment of the developing brain has been associated with unacceptable side-effects including neurocognitive deficits, potential endocrinopathies and an increased risk of secondary cancers^10^. This led to several European and North American trials aimed at avoiding or delaying radiotherapy in young children with varying degrees of success^11–15^.

Even with standard treatment protocols including radiotherapy, approximately 50% of cases still relapse. The prognosis at relapse is dismal, with only 25% of children surviving^16^. Indeed ependymoma can become a chronically relapsing disease with shortening intervals between each relapse, ultimately resulting in death. These tumours tend to invade surrounding critical structures such as the brain stem^17,18^, making complete surgical resection difficult and relapse more likely^6^.

Drug resistance is a major confounding factor in the treatment of paediatric brain tumours and ependymoma has been consistently described as a chemoresistant tumour^11,15,19,20^, although a proportion of patients with ependymoma do respond to chemotherapy regimens that achieve a high dose intensity^13,15^. Very few molecular mechanisms underlying this resistance to conventional chemotherapy are currently known^21^, hence we are unable to stratify treatment that either includes or excludes chemotherapy. There is increasing evidence, however, that a subpopulation of cancer stem cells underlies the recurrent, invasive and drug resistant nature of some tumour types^22,23^. We, and others have previously demonstrated the presence of stem-like cells in ependymomas^24,25^ and linked their existence with increased resistance to etoposide through expression of the ABCB1 multidrug transporter in stem cell enriched cell cultures^26^. Here we set out to determine the role of the multidrug efflux transporter ABCB1/P-glycoprotein in paediatric ependymoma using a panel of ependymoma derived cell lines and analyses of two clinical trial cohorts. This approach offered a unique insight into ependymoma chemotherapy resistance and the potential to identify those patients most likely to respond to chemotherapy.

## Materials and Methods

All methods were carried out in accordance with the relevant guidelines and regulations.

### Cell lines

Five paediatric ependymoma derived cell lines were used in this study (Supplementary Table S1). Whilst the EPN1^26^, EPN7 and EPN7R were established in-house; BXD-1425EPN and DKFZ-EP1 were previously characterised by Dr Xiao-Nan Li, Baylor College of Medicine^27^ and Dr Till Milde, DKFZ Heidelberg^28^ respectively. All cell lines were grown as adherent monolayers in ‘tumour media’ [15% Fetal Bovine Serum (FBS-Sigma) in 1 g/l glucose Dulbecco’s Modified Eagle Medium (DMEM-Gibco)]. DKFZ-EP1 neurospheres (DKFZ-EP1NS) were cultured in ‘stem cell media’, supplemented with growth factors^26^. C11orf95-RELA fusion gene expression, was assessed using primers designed by^8^.

### Real time PCR

Real-time PCR analysis of *ABCB1* expression was performed as previously described^29^. Relative *ABCB1*mRNA expression level was calculated using the ΔCt method^30^ and normalised with respect to *GAPDH*, which had stable transcript levels in adherent and neurosphere cultures.

### Western blotting

SDS PAGE and Western blotting were performed as previously described^29^. Blots were probed with mouse anti-ABCB1 (anti-C219 mouse monoclonal Ab; Source Bioscience 1:50), and rabbit anti-GAPDH (Cell Signaling Technology1:2000 as a loading control. Enhanced chemoluminescence (SignalFire ECL Reagent, Cell Signaling Technology) was performed according to the manufacturer’s protocol.

### Viability assays

Clonogenic assays and MTT viability assays (Cell Proliferation Kit I, Roche) were performed in order to assess response to chemotherapy in a sub-population of cells and all cells respectively. In a clonogenic assay, 600 cells/ well of the BXD-1425EPN cell line were plated in a 6-well plate and incubated with methotrexate (GeneraMedix), etoposide (Sigma) or vincristine (Sigma) in the presence or absence of the pan-ABC transporter inhibitor verapamil (20 µM) or the phosphodiesterase-5 inhibitor vardenafil (10 µM) which selectively inhibits ABCB1^31,29^. The rest of the assay was performed as per Othman *et al*.^29^, see further references therein. In the MTT assay, 6000 cells/well of both the BXD-1425EPN and the DKFZ-EP1 cell lines were plated in a 96 well–plate and, after overnight incubation for cell attachment, were incubated with the same drugs and inhibitors for a period of 5 days. Cytotoxicity was assessed using a FLUOstar plate reader (BMG lab tech instruments). The data was analysed to produce dose –response curves using Graphpad Prism Version 6.0 (GraphPad Software, La Jolla California USA).

### Wound healing assay

BXD-1425EPN and DKFZ-EP1NS cells were seeded at a density of 3 × 10^5^ and 4 × 10^5^ cells per well respectively in a 48-well plate (Corning) and cultured until the cells reached confluence before wounding^32^. Cells were treated either with vehicle (DMSO), verapamil (20 μM) or vardenafil (10 μM). Images were recorded using a Canon DS126431 camera every 4 hours for BXD-1425EPN or every 8 hours for the DKFZ-EP1 for 24 and 48 hours respectively (reflecting the different doubling times of the cell lines). The ImageJ program was used for quantifying the cell migration response by measuring the closure at 3 randomly selected positions on the wound for each condition. Wound closure curves were used to determine the t_1/2_ (time required to reach 50% closure) in Graphpad Prism Version 6. A parametric unpaired student’s t-test was used to establish any significance between treated and untreated conditions.

### 3D spheroid invasion assay

The ability of the cells to invade was assessed by carrying out a 3D spheroid assay in Cultrex Basement Membrane Extract (BME-Trevigen). 2000 cells/ well were cultured in an ultra-low attachment (ULA) 96-well round bottom plates in 100 µl tumour media, then centrifuged at 100 g for 5 minutes to encourage spheroid formation on day 4. Tumour medium was then replaced with 100 µl BME diluted to 3 mg/ml with phenol red-free RPMI-1640/1% L-glutamine on a plate warmer heated to 37 °C to facilitate BME polymerization. After 1 hour incubation 50 µl of tumour medium containing the treatment was overlaid. Images were taken daily for 4 days using a Canon DS126431 camera and were analysed using ImageJ. The relative spheroid outgrowth (R) was calculated by taking the ratio of area of the invasive edge to the area the spheroid.

### Trial cohorts and Immunohistochemistry (IHC)

The tissue microarrays (TMAs) screened in this study comprised samples from two different intracranial ependymoma clinical trial cohorts where patients had received no previous adjuvant therapy (Supplementary Table S2; Supplementary Methods). The CCLG/SIOP Infant Ependymoma clinical trial cohort (CNS9204) consisted of patients aged 3 years or under at diagnosis who were treated with chemotherapy for approximately one year with radiotherapy only given at relapse^13^. The second cohort was from the SIOP Ependymoma I clinical trial (CNS9904) and consisted of patients aged over 3 and less than 21 years at diagnosis, who were primarily treated with radiotherapy with chemotherapy only given if, after a second attempt at surgery, resection remained incomplete. These studies, and the experimental protocols required, were reviewed and approved by the National Research Ethics Service Committee East Midlands – Nottingham 2 and have therefore been performed in accordance with the ethical standards laid down in an appropriate version of the 1975 Declaration of Helsinki, as revised in 1983. For all patients, informed consent was obtained from the patient, or a parent and/or legal guardian where the patient was under 18 years of age, prior to their inclusion in the study.

IHC staining was performed using the mouse anti-ABCB1 monoclonal antibody (C219, Millipore) at concentration of 1:40. The Dako Chemate Envision Antigen Detection kit (Dako, UK) was used as described by Othman *et al*.^29^. The association between immunohistochemical status and overall survival (OS) as well as event free survival (EFS) was investigated using the Kaplan–Meier method, with differences estimated using the long-rank (Mantel–Cox) test. OS was defined as the time between the date of diagnosis and death whilst EFS was defined as the time between date of diagnosis and date of first event (recurrence/death). Patients still alive at the end of the study were censored at the date of the last follow-up. The effect of multiple confounding factors were analysed by the Cox proportional hazard regression model to determine the robustness of ABCB1 as an independent predictor of survival. Data analyses were performed with IBM SPSS 22.0 for Windows (IBM Corp. Armonk, NY, USA).

## Results

### ABCB1 inhibition in ependymoma cell lines potentiates the effect of chemotherapy

**Figure 1:**
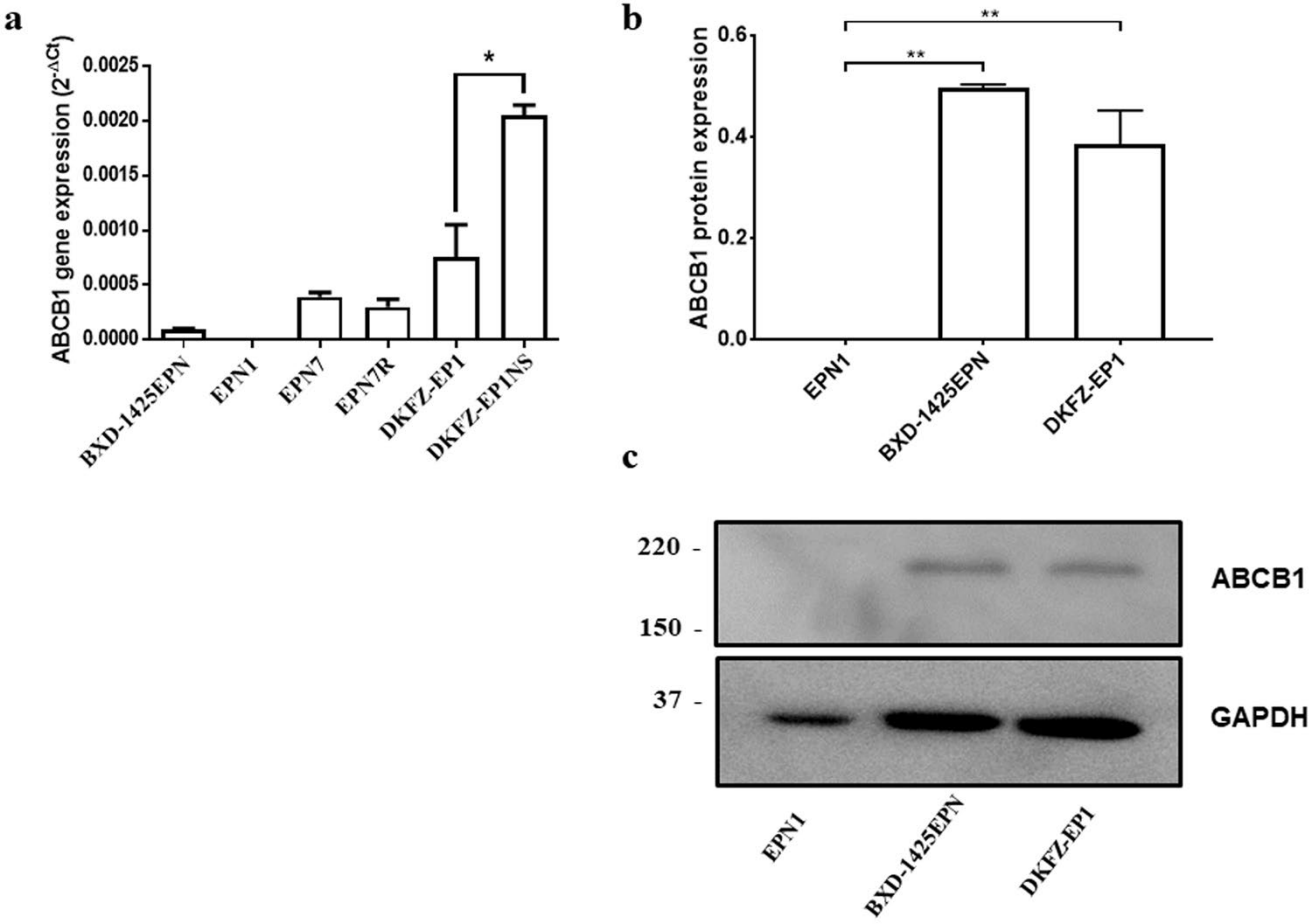
ABCB1 is expressed in paediatric ependymoma derived cell lines. (**a**) ABCB1 gene expression relative to the GAPDH was calculated for each cell line using the ΔCt method^30^. 4 out 5 ependymoma cell lines expressed ABCB1. An approximately 3 fold increase in ABCB1 expression was observed when the DKFZ-EP1 cell line was enriched for stemness by growing it as neurospheres (DKFZ-EP1NS). (**b**) Expression of ABCB1 protein was analysed by western blotting in 20 µg of protein isolated from EPN1, BXD-1425EPN and DKFZ-EP1 and quantified relative to GAPDH as a loading control. c. A representative western blot showing ABCB1 and GAPDH expression. *p ≤ 0.05, **p ≤ 0.01.

*ABCB1* gene expression was investigated in 3 previously published (EPN1^26^, BXD-1425EPN^27^ and DKFZ-EP1^28^) and 2 newly established (EPN7 and EPN7R) ependymoma cell lines. Sequence analysis revealed that, apart from EPN7/7R, these lines harboured a C11orf95-RELA fusion gene (Supplementary Table S1) making them representative of ST-EPN-RELA aggressive subgroup of ependymomas known to respond poorly to current therapies^8^. In comparison to the GAPDH housekeeping gene, 4 out of 5 of these lines demonstrated relatively low but consistent expression of *ABCB1* (Fig. 1a). In common with our previously published findings in medulloblastoma we found that enriching for stemness in neurosphere culture resulted in a 3 fold increase in *ABCB1* expression (Fig. 1a). *ABCB1* expression was also shown to be maintained at recurrence in cultured cells since EPN7 and EPN7R were derived from primary and recurrent tumours from the same patient. Expression of ABCB1 protein was confirmed in BXD-1425EPN and DKFZ-EP1 whereas expression was not observed in EPN1 (Figs 1b,c and S1).

**Figure 2:**
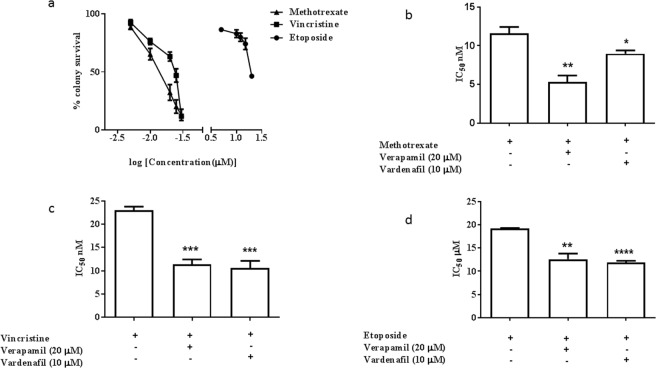
ABCB1 inhibition potentiated response to chemotherapy in BXD-1425EPN. (**a**) Cytotoxic response of the BXD-1425EPN cell line to the chemotherapeutic drugs methotrexate, vincristine and etoposide was assessed by the stem cell relevant clonogenic assay to produce dose response curves (100% represents vehicle control). (**b–d**) The IC_50_ concentrations for each drug were then recalculated in the presence of either the non-specific ABCB1 inhibitor verapamil (20 µM) or the selective ABCB1 inhibitor, vardenafil (10 µM). There was a significant potentiation of cytotoxic response represented by reduction in the IC_50_ concentrations of methotrexate (**b**), vincristine (**c**) and etoposide (**d**) when combined with either inhibitor (*p ≤ 0.05, **p ≤ 0.01, ***p ≤ 0.005, **** p ≤ 0.001).

Downstream functional analyses were carried out on two robustly growing ABCB1 expressing cell lines; BXD-1425EPN and DKFZ-EP1. Clonogenic survival was assessed in BXD-1425EPN in response to three ABCB1 substrates that are commonly used in the treatment of paediatric ependymoma. We found that whilst these cells were sensitive to methotrexate and vincristine in the nanomolar range (IC_50_ 12 nM and 23.5 nM respectively), micromolar concentrations of etoposide were required to elicit a response (IC_50_ = 20 μM) (Fig. 2a). Both verapamil (a calcium channel blocker that inhibits several ABC transporters) and vardenafil (a phosphodiesterase 5 inhibitor, which specifically inhibits ABCB1) were able to significantly potentiate the cytotoxic effect of all 3 drugs in clonogenic (Figs 2b–d and S2) and MTT cell viability assays (Fig. S3). In contrast, we were unable to obtain an IC_50_ value for any of the 3 drugs in MTT assays with the DKFZ-EP1 cell line (Fig. S4). We did observe a slight potentiation in cytotoxic response to methotrexate when these cells were co-treated with verapamil; although at extremely high (clinically unachievable) drug concentrations.

### ABCB1 inhibition reduced migration and invasion of ependymoma cells

**Figure 3:**
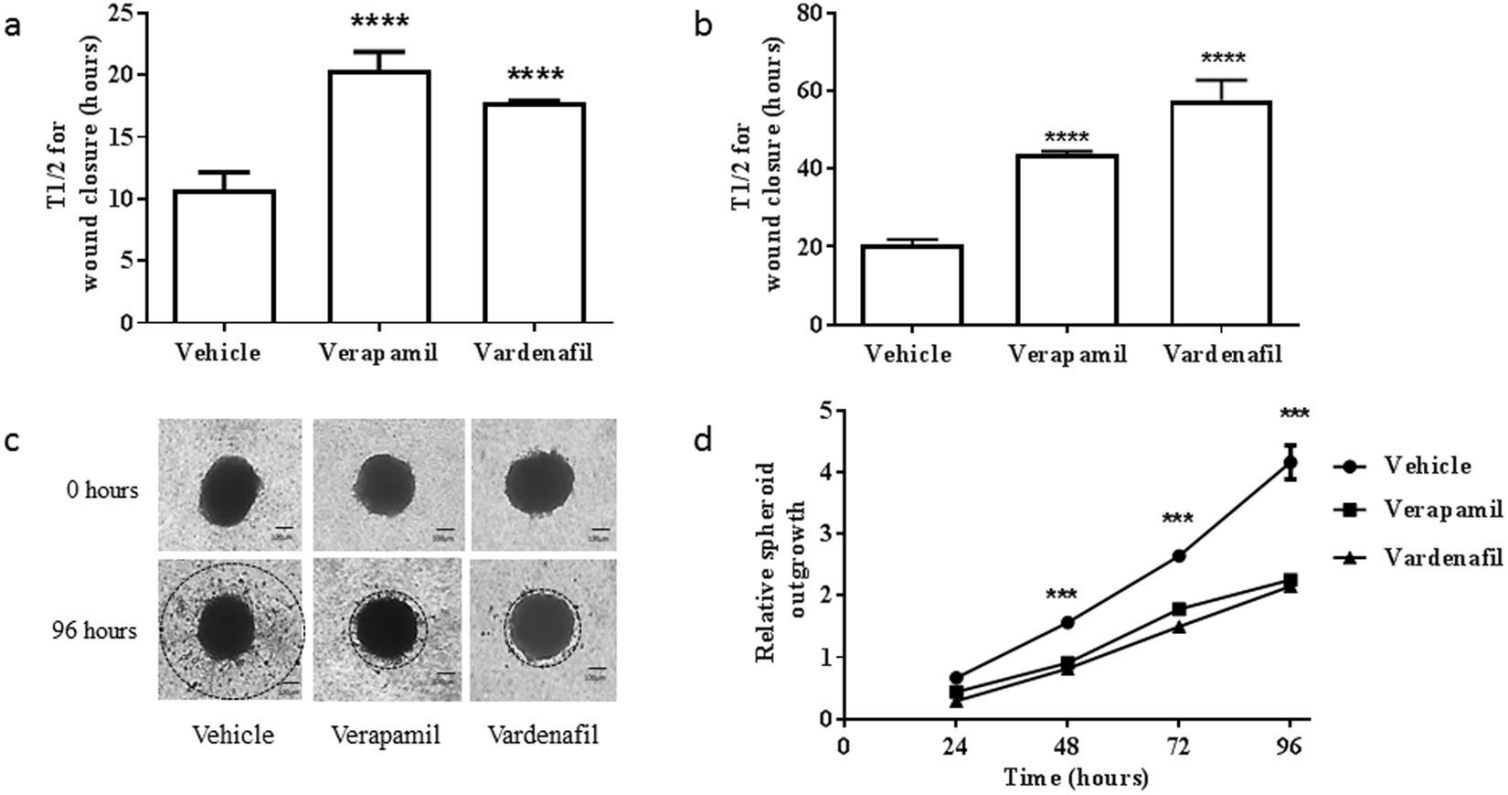
ABCB1 inhibition reduces migration and invasion in ependymoma cell lines. The ability of BXD1425EPN (**a**,**c**,**d**) and DKFZ-EP1 (**b** & Fig. S5) cells to migrate and invade was measured in wound healing (**a**,**b**) and spheroid invasion (**c**,**d**) assays respectively. The time required for 50% wound closure (t_1/2_) was significantly increased when both the BXD-1425EPN (**a**) and DKFZ-EP1 (**b**) cell lines were treated with either the non-specific ABCB1 inhibitor, verapamil (20 µM) or the specific ABCB1 inhibitor, vardenafil (10 µM). (**c**) A 3D spheroid invasion assay was performed to assess the ability of ependymoma cells to digest and invade through extracellular matrix (Cultrex BME). The spheroids formed from BXD-1425EPN cell line formed invadopodia and demonstrated multicellular streaming, which was visibly reduced after 96 hours in the presence of either ABCB1 inhibitor. (**d**) There was a significant reduction in the relative spheroid outgrowth R (ratio of the area of the invasive edge [dotted line] to the area of the area of spheroid itself) when treated with either ABCB1 inhibitor, which became more pronounced at 96 hours. Scale bars represent 100 µm. ***p ≤ 0.005, ****p ≤ 0.001.

ABCB1 has also been proposed to play a role in cell migration^33–36^. BXD-1425EPN formed an invasive tumour in mouse orthotopic xenografts^27^ and DKFZ-EP1 was derived from pleural ascites of a metastatic ependymoma^28^ indicating that both would be good models to test in cell migration assays. In vehicle-only treated control experiments, wound closure occurred with a t_1/2_ of 11 and 20 hours for the BXD-1425EPN and the DKFZ-EP1 cell line respectively. Faster wound closure for BXD-1425EPN may reflect the slightly higher ABCB1 protein levels in this cell line (Fig. 1b,c). The t_1/2_ for wound closure was significantly increased (p ≤ 0.001) in both cell lines in the presence of either verapamil or vardenafil (Fig. 3a,b), supporting the hypothesis that ABCB1 function plays a role in cell migration. In spheroid invasion assays, BXD-1425EPN cells were able to readily invade through extracellular matrix, a process which was significantly inhibited (p ≤ 0.005) by the addition of either of the inhibitors (Fig. 3c,d). In contrast, DKFZ-EP1 cells formed circumscribed spheroids which failed to invade through BME (Fig. S5).

### ABCB1 protein expression was independently associated with reduced overall and event free survival in a chemotherapy led trial

**Figure 4:**
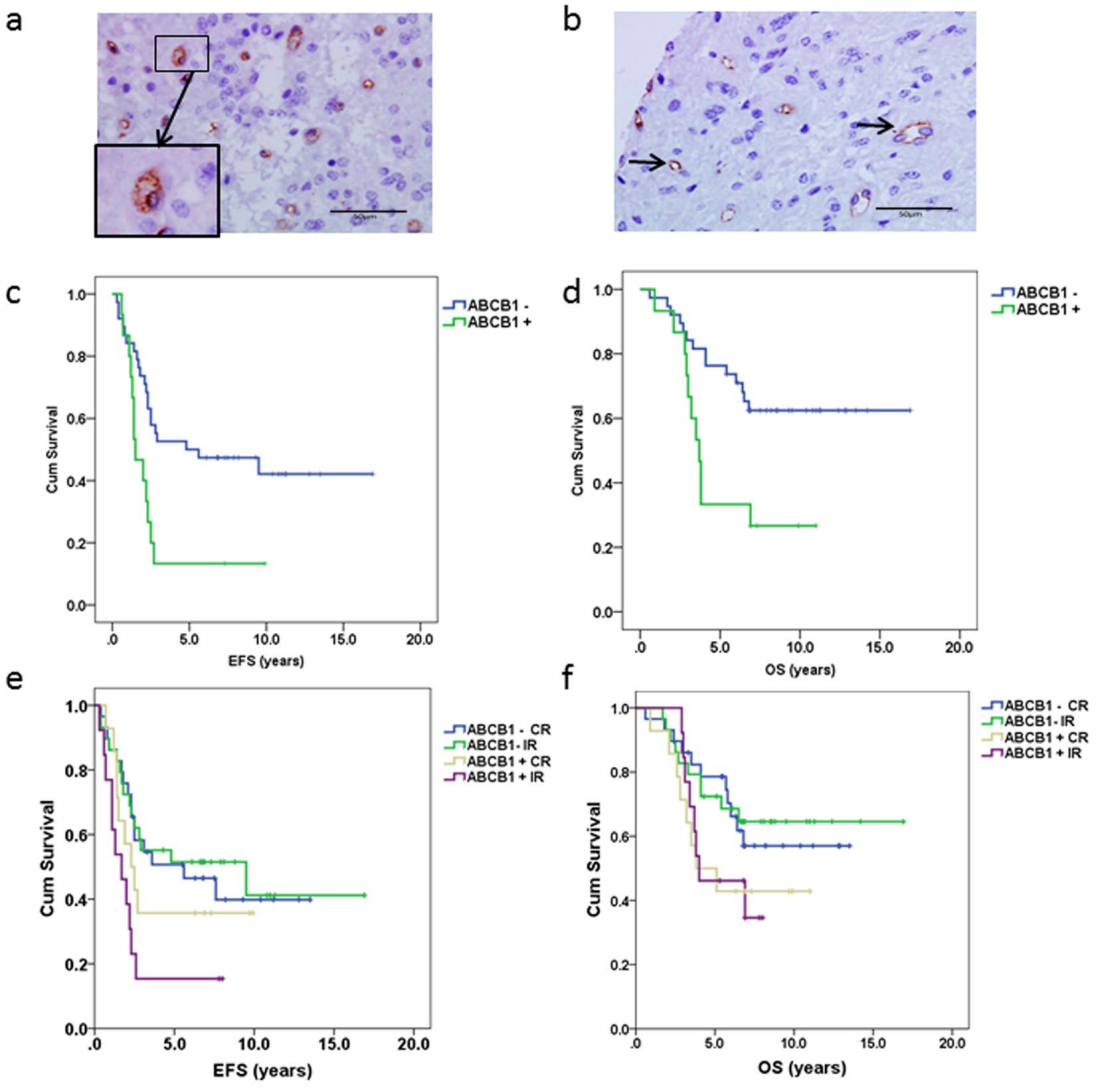
Membranous expression of ABCB1 was associated with poor survival and early relapse in ependymoma. Tissue microarrays from the CNS9204 clinical trial cohort were screened for ABCB1 protein expression. (**a**) An ependymoma patient sample in which a sub-population of tumour cells stained positive for membranous ABCB1 expression (boxed and magnified). (**b**) Ependymoma samples which demonstrated vascular staining (arrows) in the absence of membranous staining tumour cells were scored as negative. Scale bars represent 50 µm. (**c**) ABCB1 positive patients from the chemotherapy-led (CNS9204) trial had a significantly reduced event-free survival (5-year EFS 13% vs. 50%, p = 0.007). (**d**) Overall survival was also significantly reduced in ABCB1 positive CNS9204 patients (5-year OS 33% vs. 74%, p = 0.009) respectively. Data from both trials were combined in order to explore the potential effect modification between ABCB1 and resection status. **e**. Patients with an incomplete resection who were ABCB1 positive had the poorest EFS (5-year EFS- 15%, p = 0.03) in comparison to other groups. (**f**) Overall survival was not significantly correlated with resection status although patients who were ABCB1 positive had the worst prognosis. CR complete resection, IR incomplete resection, +censored.

In order to address the hypothesis that ABCB1 was functioning as a multidrug transporter in a subpopulation of ependymoma cancer stem cells, we compared outcome in paediatric patients from a primary infant chemotherapy trial (CNS9204) to those from a primary radiotherapy trial (CNS9904). Tumour samples were scored as positive or negative for membranous ABCB1 expression using immunohistochemistry (Fig. 4a,b respectively; tumours with only vascular staining were scored as negative). Positive expression, where observed, was always in less than 3% of cells (median 0.33%). In total, 27 of 85 primary tumours (32%) were scored as positive for ABCB1 by standard immunohistochemical analysis, 15/53 (28%) from the CNS9204 trial cohort and 12/32 (37.5%) from the CNS9904 cohort. In the CNS9204 trial cohort, outcome for ABCB1 positive-patients correlated with lower 5 year event free survival (EFS; 13% versus 50%) P = 0.007 (Fig. 4c) and lower 5 year overall survival (OS; 33% versus 74%) p = 0.009 (log-rank analysis) (Fig. 4d). The correlation with outcome held in Cox Regression multivariate analysis (Table 1) where ABCB1 was an independent factor, even after adjustment for resection status and grade, for EFS (Hazard Ratio 2.8 confidence interval 1.3–5.9) p = 0.007 and OS (Hazard Ratio 3.0 confidence interval 1.3–6.8) p = 0.008. In the primary radiotherapy trial CNS9904 cohort there was no correlation between ABCB1 expression and outcome for EFS or OS by log-rank analysis (Fig. S6).

**Table 1:** Multivariate analysis of ABCB1 expression in the chemotherapy-led CNS9204 trial.

Survival	Factor	Hazard Ratio (95% confidence interval)	P value
Event-Free Survival (EFS)	ABCB1 expression (Positive vs. Negative)	2.79 (1.33–5.87)	0.007
	Resection (Incomplete vs. Complete)	1.32 (0.67–2.74)	0.39
	WHO Grade (Grade III Vs. Grade II)	1.06 (0.54–2.12)	0.84
Overall Survival (OS)	ABCB1 expression (Positive vs. Negative)	3.0 (1.32–6.81)	0.008
	Resection (Incomplete vs. Complete)	1.32 (0.58–3.0)	0.71
	WHO Grade (Grade III vs. Grade II)	1.16(0.52–2.60)	0.49

### ABCB1 protein expression was associated with early relapse in *all* patients with incomplete resection

Whilst patients in the CNS9904 trial were primarily treated with radiotherapy, a subset of these patients, where tumour resection was deemed incomplete, were also given chemotherapy. In order to assess the prognostic value of ABCB1 in all patients who received chemotherapy and explore the potential effect modification by tumour resection status, we combined the two trial datasets. As shown in Fig. 4e, EFS curves differ across the four subgroups (log rank test p = 0.03). Compared to ABCB1 negative patients with complete resection, ABCB1 positive patients are more likely to relapse, in particular those who had incomplete tumour resection (hazard ratio 2.64, 95% CI 1.22–5.73). This effect remained significant after further adjustment for age, WHO tumour grade and whether or not patients received radiotherapy (adjusted HR 2.9, 95% CI 1.3–6.5, p = 0.008). The overall survival for all ABCB1 positive patients was poor regardless of the resection status (Fig. 4f). Although none of the subgroups reached significance, ABCB1 positive patients in general showed an increased risk of death (HR = 1.85, 95% CI 0.74–4.59 and HR 1.82, 95% CI 0.73–4.55 for patients with complete and incomplete resection, respectively), compared with ABCB1 negative patients.

ABCB1 status could be correlated with methylation subgroup for 36 of the patients included in this study. ABCB1 was expressed in 3 subtypes (EPN PFA, ST-EPN-RELA and EPN PFB), although percentages are only meaningful in the EPN PFA (30%; 8/26) and the ST-EPN-RELA subgroups (43%; 3/7). Log rank analysis indicated that although ABCB1 positive patients appeared to do less well in the PFA subgroup this did not reach significance indicating that ABCB1 expression was a confounding factor across both subgroups.

## Discussion

The aim of this study was to investigate whether the presence of a sub-population of multidrug transporter expressing cancer cells could play a role in promoting relapse and progression of ependymomas in response to postoperative chemotherapy. Uniquely, we were able to investigate multidrug transporters in a cohort of patient samples from the same primary chemotherapy trial and compare outcome to samples from a primary radiotherapy trial. Thus, we were also able to correlate expression with the specific type of chemotherapy that the patients received. Notably, 3 out of the 5 chemotherapy drugs methotrexate^37^, vincristine^37^ and cyclophosphamide^38^ used to treat this cohort are ABCB1 substrates. In the primary chemotherapy-led trial cohort, ABCB1 positivity was an independent prognostic factor for EFS, with ABCB1-positive patients having significantly shorter mean EFS of just 2.7 years compared to their ABCB1 negative counterparts who took a mean of 8.6 years to experience an event. This observation comes despite tumour positivity for ABCB1 being heterogeneous; indeed ABCB1 positivity was no higher than 3% of cells. Hence, ABCB1 expression in a small sub-population of tumour cells is able to confer a drug resistant phenotype in paediatric ependymoma patients in CNS9204.

ABCB1 expression was just as prevalent in the radiotherapy cohort as the primary chemotherapy cohort (37.5% and 28% positive tumours respectively) suggesting that ABCB1 associated drug resistance is not unique to very young children (those under 3 years) but that it is the clinical preference for radiotherapy avoidance that makes it a strong prognostic factor in the younger age group. This was supported by our finding that ABCB1 expression correlated with poorer event free survival across all patients who received chemotherapy. Together, these novel prognostic associations have two implications for ependymoma therapy for the 0–19 age group, stratified according to ABCB1 status. Firstly, there is the possibility that radiotherapy, with its associated increased risk of secondary tumours and effects on the developing brain at all ages of childhood, could be avoided in ABCB1 negative tumours. Furthermore, ABCB1 inhibition may increase the efficacy of adjuvant chemotherapy in almost a third of paediatric ependymomas (in total 32% of patients were ABCB1 positive over both trials). Another potential advantage of ABCB1 inhibition would be increased drug uptake at the blood-tumour barrier, since we have also been able to detect ABCB1 expression in the blood vessels supplying ependymomas (Fig. 4b). ABCB1 expression in endothelial cells also restricts drug access to the brain at the blood brain barrier, limiting the exposure of brain tumours to systemic chemotherapy^39^. Thus, inhibition of ABCB1 alongside chemotherapy may be of benefit to all patients, by increasing drug uptake as well as rendering previously therapy-resistant tumours sensitive to ABCB1 substrates. This may also be beneficial in allowing drugs to be administered at lower concentrations with reduction in associated toxicity.

As as well as its role in drug resistance, ABCB1 has been proposed to play a role in cell migration^33–36^. Notably, local invasion remains a clinical marker of poor survival in ependymoma^6,18^ and therefore the inhibition of ABCB1 in a non-toxic, specific manner may be relevant in both contexts. The paucity of representative cell line models that retain a sub-population of ABCB1 expressing cancer stem cells and are amenable to pre-clinical assays has hampered progress towards this goal. To this end, we have been able to demonstrate that 4 out of the 5 ependymoma derived cell lines tested expressed ABCB1 by quantitative PCR. ABCB1 expression levels could be enriched by neurosphere culture thus supporting its expression in a subpopulation of cancer stem cells. Using ABCB1 inhibitors alone we were able to significantly reduce the migration of ependymoma cells in a wound healing assay (p ≤ 0.001) in both cell lines. Invasion of BXD1425-EPN spheroids into basement membrane extract was also significantly impeded by ABCB1 inhibition (p ≤ 0.001). BXD-1425EPN showed a response to methotrexate and vincristine within a clinically achievable range and this sensitivity was heightened by inhibition with the ABCB1 specific inhibitor vardenafil or the non-specific ABC transporter inhibitor verapamil. In contrast the high ABCB1 expressing cell line DKFZ-EP1 proved highly resistant to all three chemotherapeutics. DKFZ cells were derived from a highly aggressive supratentorial anaplastic grade III ependymoma, which has previously been demonstrated to be highly resistant to chemotherapeutic agents including vincristine, cisplatin and temozolomide^28^. The inability to reverse this resistance indicates that other mechanisms in addition to ABCB1 are operating in this highly aggressive cell line.

In summary, we have shown that, ABCB1 expression in a sub-population of cells is correlated with poorer outcome and survival, presumably reflecting a sub-set of tumours that are both chemoresistant and locally invasive across the two clinical trial cohort assessed. On this basis ABCB1 is one of the key biomarkers being evaluated in the ongoing SIOP ependymoma II trail through the BIOMECA consortium. In cell line models that recapitulate ABCB1 expression, drug resistance and invasion can be decreased by ABCB1 inhibitors. ABCB1 expression might therefore provide a mechanism by which a patient’s likelihood of responding to chemotherapy is assessed so that chemotherapy can be reserved for those most likely to respond. In addition, the ability to potentiate chemotherapy and to reduce local tumour invasion would be a significant advance and this work implicates ABCB1 inhibition with vardenafil, a repurposed paediatric-compatible drug^40^ as being a potential mechanism to achieve this.

## Supplementary information

Supplementary information

## Data Availability

All data generated or analysed during this study are included within the article (and its Supplementary Information files).
