# Supplementary material for: A role for ABCB1 in prognosis, invasion and drug resistance in ependymoma
Source: Sci Rep. 2019 Jul 16;9:10290. doi: 10.1038/s41598-019-46700-z (PMC6635358; doi:10.1038/s41598-019-46700-z)
Supplement: Supplementary file 1 — Supplementary information [file 41598_2019_46700_MOESM1_ESM.pdf]

A role for ABCB1 in prognosis, invasion and drug resistance in ependymoma

Durgagauri H. Sabnis, Lisa C.D. Storer, Jo-Fen Liu, Hannah K. Jackson, J.P Kilday, Richard G. Grundy, Ian D. Kerr, Beth Coyle

Supplementary Figures S1-S6

Supplementary Tables S1 and S2

Supplementary materials and methods (trial cohorts)

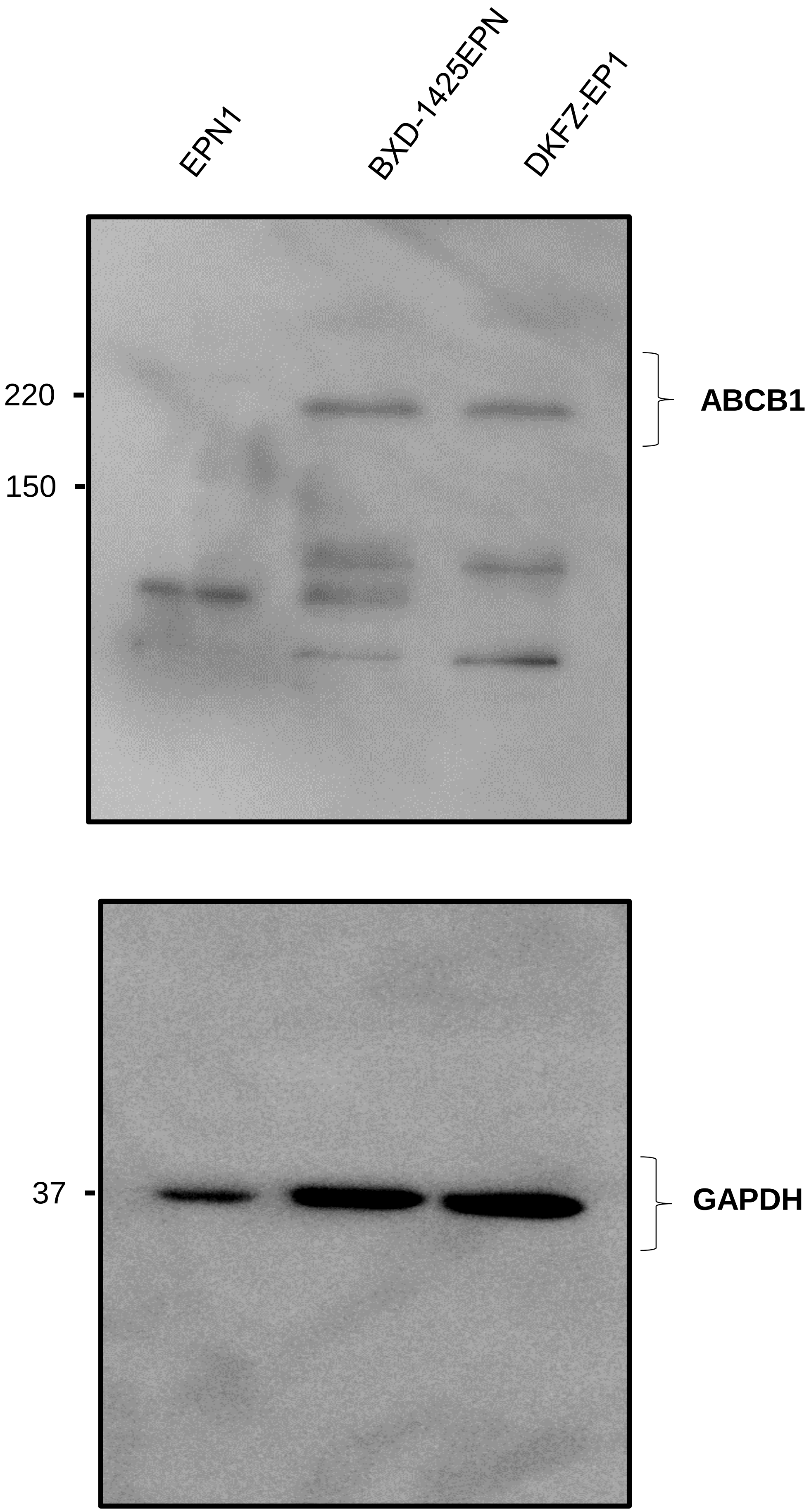

Fig. S1 ABCB1 expression in ependymoma Uncropped blots showing expression of ABCB1 protein was analysed by western blotting in 20 µg of protein isolated from EPN1, BXD-1425EPN and DKFZ-EP1 with GAPDH as a loading control.

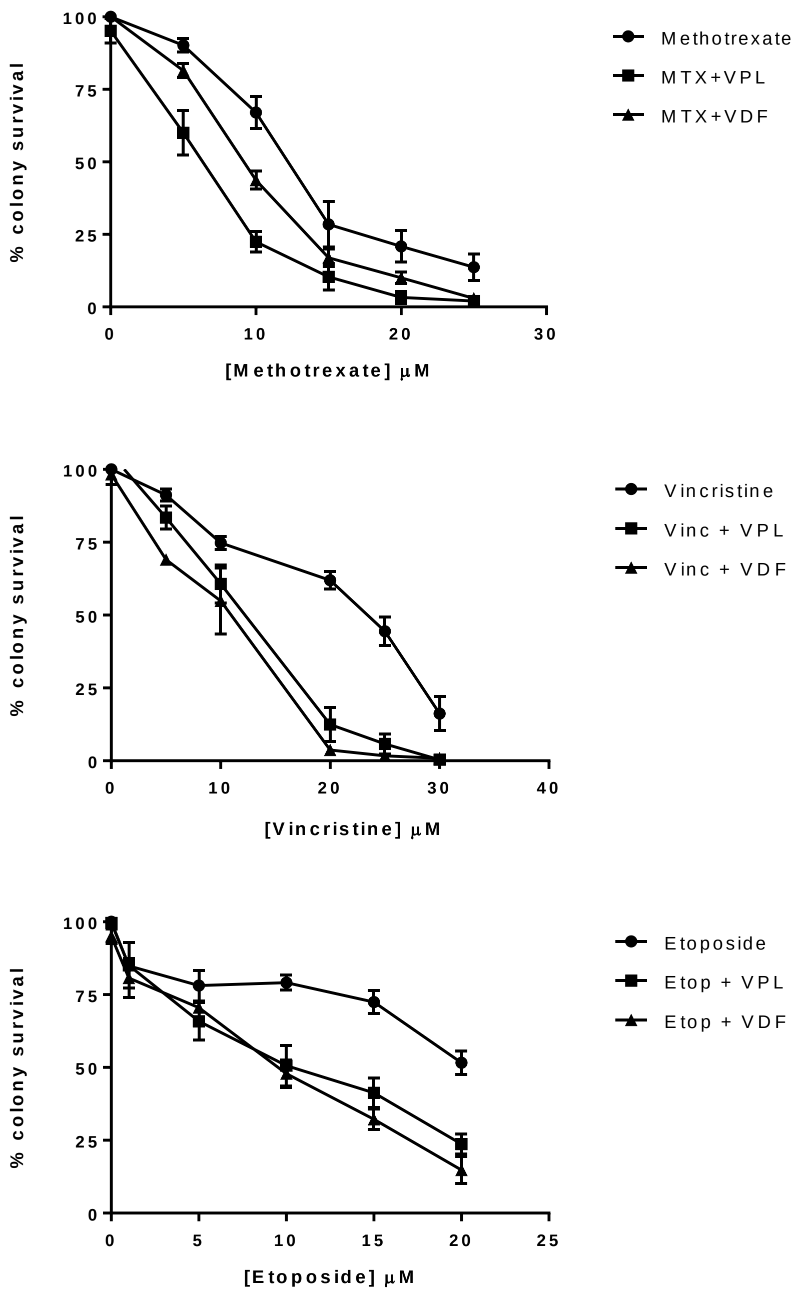

Fig. S2 ABCB1 inhibition potentiated response to chemotherapy in ependymoma BXD-1425EPN cell line in the clonogenic assay Cytotoxic response of BXD-1425EPN cell line to chemotherapeutic drugs methotrexate**,** vincristine and etoposide was measured by the clonogenic assay. In each case a leftward shift of the dose-response curves was observed when vardenafil or verapamil were combined with cytotoxic drugs (methotrexate, etoposide and vincristine). In the absence of cytotoxic drugs (0μM) , the applied concentrations of verapamil and vardenafil are not toxic as colony survival in these cases is 100%.

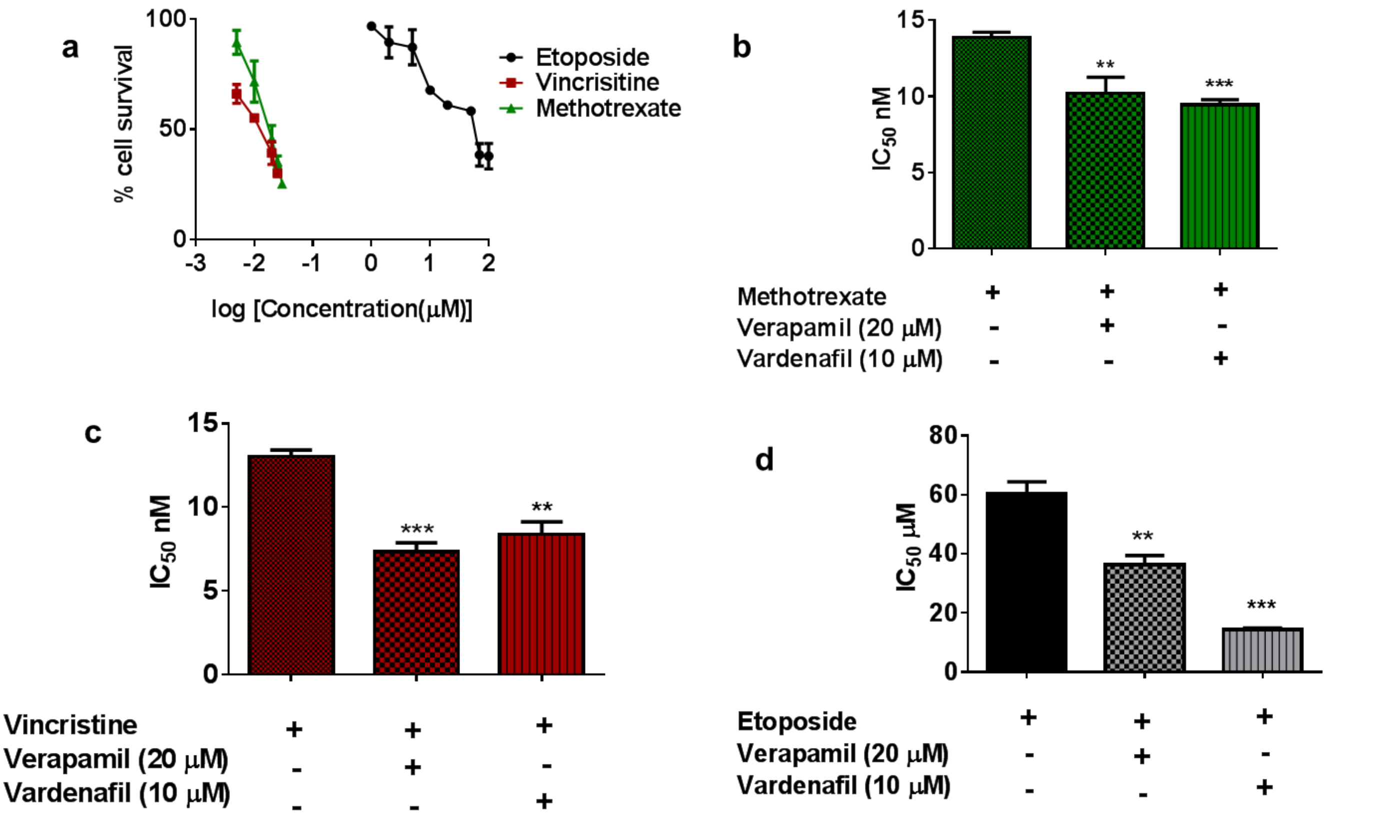

Fig. S3 ABCB1 inhibition potentiated response to chemotherapy in ependymoma BXD-1425EPN cell line by the MTT assay a Cytotoxic response of BXD-1425EPN cell line to chemotherapeutic drugs methotrexate**,** vincristine and etoposide was measured by the MTT assay. **b-d.** There was a significant potentiation of cytotoxic response depicted by a reduction in the IC_{50} concentrations of the drugs when combined with both the non-specific ABCB1 inhibitor verapamil (20μM) and the selective ABCB1 inhibitor, vardenafil (10μM). **p≤0.01***p≤0.005

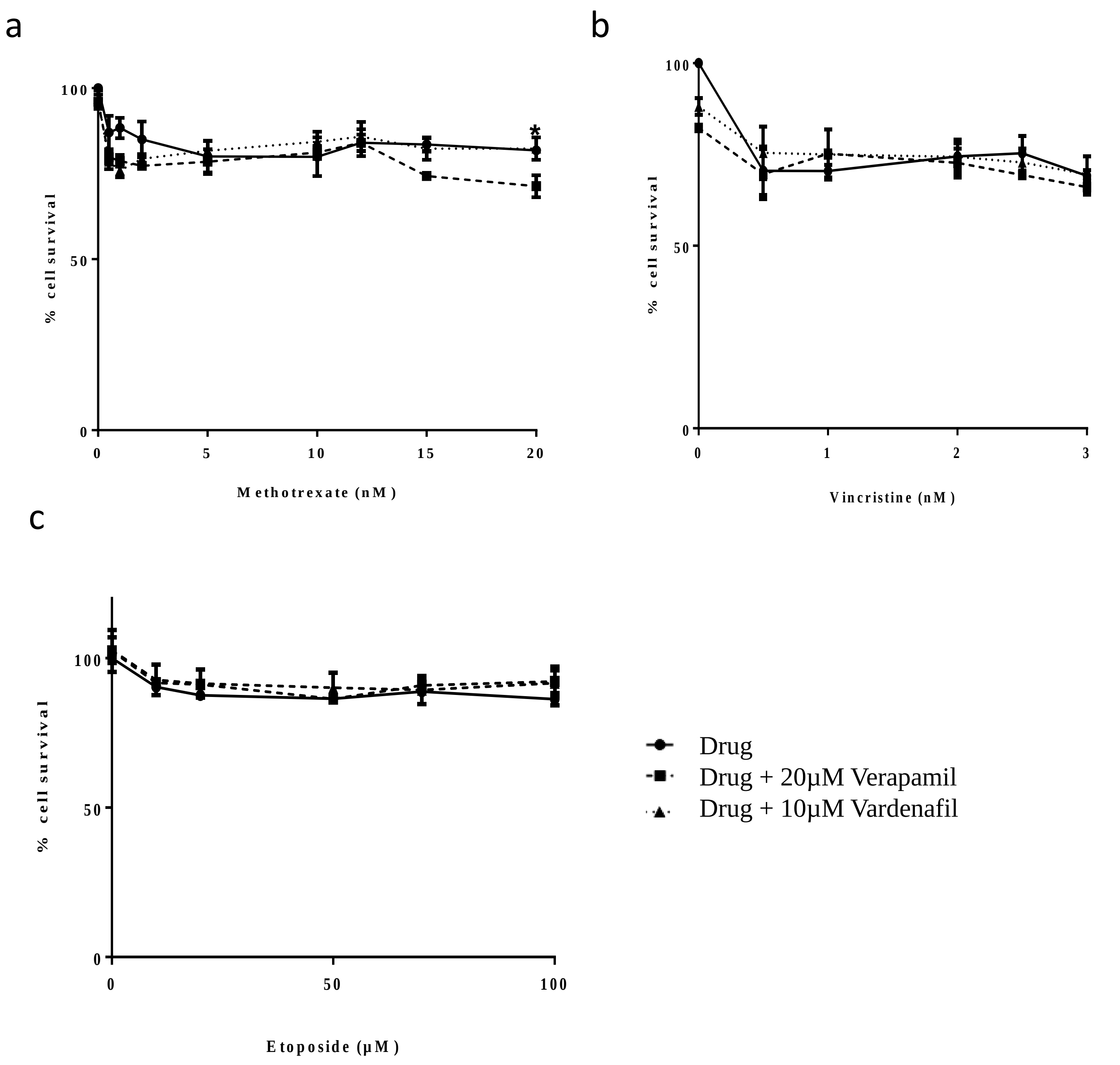

Fig. S4 The DKFZ-EP1 ependymoma cell line was resistant to standard chemotherapy Since the DKFZ-EP1 cell line did not form colonies, cytotoxic response to the three chemotherapeutic drugs was assessed by the MTT assay. **a-c.** DKFZ-EP1 cells exhibited resistance to methotrexate, vincristine and etoposide, with the IC50 not being reached over a wide concentration range. There was a marginal potentiation of response to methotrexate (**a**; *$p \leq 0.05$) at the highest concentration of drug tested when combined with verapamil (20 µM) while no potentiation was recorded with etoposide or vardenafil (10 µM).

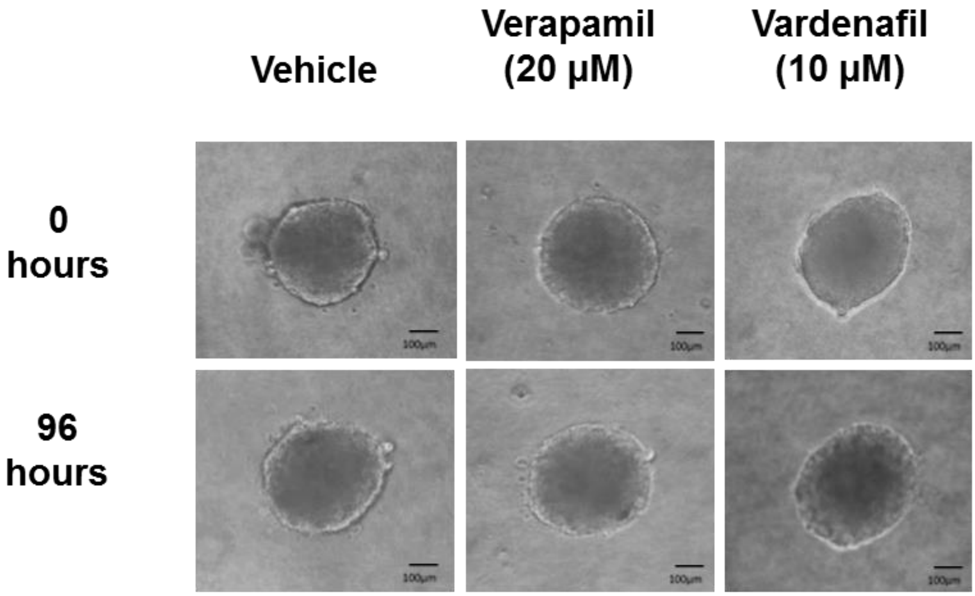

Fig. S5 DKFZ-EP1 cells did not invade in 3-D culture A 3D spheroid invasion assay was performed to assess the effect of ABCB1 inhibition on the ability of DKFZ-EP1 cells to digest and invade through extracellular matrix (Cultrex BME). Spheroids formed from the DKFZ-EP1 cell line remained circumscribed in a 3D invasion assay and showed no change in phenotype over the length of the assay (96 hours) regardless of treatment. Scale bars in the images represent 100 µm.

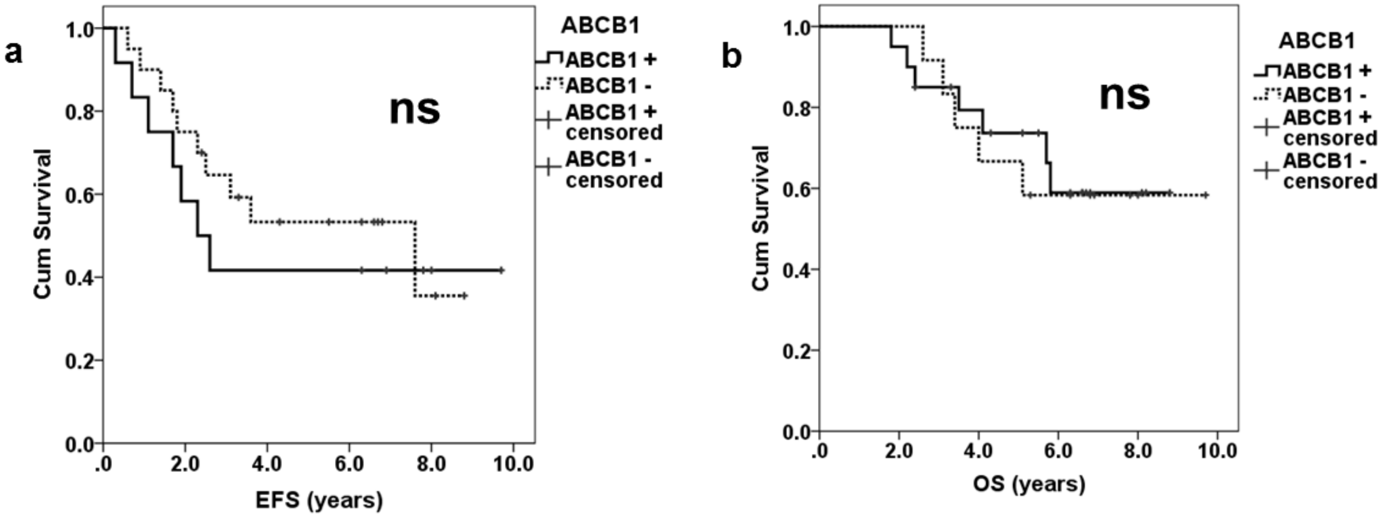

Fig. S6 Membranous ABCB1 expression did not correlate with survival in the radiotherapy–led CNS9904 trial Tissue microarrays from the CNS92904 clinical trial cohort were screened for ABCB1 protein expression. **a.** ABCB1 positive patients from the radiotherapy-led (CNS9904) trial had similar event free survival to their ABCB1 negative counterparts (p=0.578). **b.** Similarly, overall survival was unaffected by ABCB1 status in patients primarily treated with radiotherapy (p=0.861).

Supplementary Table S1 Clinicopathological details of cell lines.

Cell line	Patient details	WHO Grade	Molecular grade
BXD-1425EPN (Baylor - Xenograft Derived)	9 year old male	Recurrent supratentorial anaplastic Grade III ependymoma	ST-EPN-RELA[a]
EPN1	22 year old male	Recurrent supratentorial grade II ependymoma	ST-EPN-RELA[a]
EPN7	3 months female	Primary supratentorial grade III ependymoma	-
EPN7R	18 months female	Recurrent supratentorial grade II ependymoma	-
DKFZ-EP1	18 year old female	Recurrent supratentorial anaplastic Grade III ependymoma (peritoneal metastasis)	ST-EPN-RELA[a]

[a] Defined by sequence analysis

Supplementary Table S2 Clinicopathological characteristics of paediatric ependymoma patient samples assessed across two European trial cohorts, CCLG (formerly UKCCSG) 1992 04(CNS9204) and SIOP 1999 04 (CNS9904)

Variable		CNS9204 (n=60)	CNS9904 (n=47)	P value
Gender	Male	39 (65 %)	20 (43 %)	0.0021
	Female	21 (35 %)	27 (57 %)	
Resection Status	Complete	28 (47 %)	23 (49 %)	0.84
	Incomplete	32 (53 %)	24 (51 %)	
Grade	WHO primary tumour Grade II	33 (55 %)	28 (60 %)	0.69
	WHO primary tumour Grade III	27 (45 %)	19 (40 %)	
Tumour Location	Posterior fossa (PF)	53 (88 %)	28 (60 %)	0.001
	Supratentorial (ST)	7 (12 %)	19 (40 %)	
Age	< 3 years	59 (98 %)	2 (4 %)	<0.001
	> 3 years	1 (2 %)	45 (96 %)	
Status	Dead	29 (48 %)	19 (40 %)	0.31
	Alive	31 (52 %)	28 (60 %)	
Event occurred (relapse/ death)	Event	40 (67 %)	26 (55 %)	0.31
	No event	20 (33 %)	21 (44 %)	

Supplementary materials and methods

Trial cohorts

The CCLG/SIOP Infant Ependymoma clinical trial cohort (CNS9204) consisted of patients aged 3 years or under at diagnosis. Treatment involved maximal resection followed by 4 courses of alternating myelosuppressive and non-myelosuppressive chemotherapy (vincristine, carboplatin, methotrexate, cyclophosphamide and cisplatin) at 14 day intervals for a total of 7 cycles lasting approximately 1 year in duration. Radiotherapy was only given to these patients if progressive disease (relapse) was identified by neuro-imaging. Of the 89 patients recruited to this study, 60 samples were available for TMA analysis of which 53 were scorable due to core loss. The second cohort was from the SIOP Ependymoma I clinical trial (CNS9904) and consisted of patients aged over 3 and less than 21 years at diagnosis. Treatment for these patients involved either complete resection followed by adjuvant radiotherapy to the primary tumour site within 4 weeks of a complete resection, or in cases of incomplete resection, followed by further attempted second surgery then focal radiotherapy or radiotherapy alone or combined with four cycles of chemotherapy (vincristine, etoposide and cyclophosphamide) if resection still remained unachieved. Of the 79 ependymoma patients (89 were originally enrolled, 10 removed due to misdiagnosis) included in this study, 47 samples were available for tissue microarray analysis of which 32 were scorable due to core loss. There was no significant difference in distributions of gender, resection status, tumour grade, location and event rate between sub-cohorts and trial cohorts. Between the trial cohorts, there was no significant difference in distributions of gender, resection status, tumour grade, location and significant differences, apart from age and axiomatic treatment, were tumour location (p=0.001) with the majority being infratentorial in the younger CNS9204 trial cohort, and gender (p=0.021) where there were almost double the incidence of males in the CNS9204 samples.
